# Glycerol metabolism impacts biofilm phenotypes and virulence in *Pseudomonas aeruginosa* via the Entner-Doudoroff pathway

**DOI:** 10.1128/msphere.00786-23

**Published:** 2024-03-19

**Authors:** Somalisa Pan, Simon A. M. Underhill, Christopher W. Hamm, Mylissa A. Stover, Daxton R. Butler, Crystal A. Shults, Jacob R. Manjarrez, Matthew T. Cabeen

**Affiliations:** 1 Department of Microbiology, Oklahoma State University, Stillwater, Oklahoma, USA; 2 Department of Biochemistry and Microbiology, OSU Center for Health Sciences, Tulsa, Oklahoma, USA; The University of Iowa, Iowa City, Iowa, USA

**Keywords:** biofilm, glycerol, *Pseudomonas*, infection, Entner-Doudoroff, metabolism

## Abstract

*Pseudomonas aeruginosa* is a ubiquitous bacterium and a notorious opportunistic pathogen that forms biofilm structures in response to many environmental cues. Biofilm formation includes attachment to surfaces and the production of the exopolysaccharide Pel, which is present in both the PAO1 and PA14 laboratory strains of *P. aeruginosa*. Biofilms help protect bacterial cells from host defenses and antibiotics and abet infection. The carbon source used by the cells also influences biofilm, but these effects have not been deeply studied. We show here that glycerol, which can be liberated from host surfactants during infection, encourages surface attachment and magnifies colony morphology differences. We find that glycerol kinase is important but not essential for glycerol utilization and relatively unimportant for biofilm behaviors. Among downstream enzymes predicted to take part in glycerol utilization, Edd stood out as being important for glycerol utilization and for enhanced biofilm phenotypes in the presence of glycerol. Thus, gluconeogenesis and catabolism of anabolically produced glucose appear to impact not only the utilization of glycerol but also glycerol-stimulated biofilm phenotypes. Finally, waxworm moth larvae and nematode infection models reveal that interruption of the Entner-Doudoroff pathway, but not abrogation of glycerol phosphorylation, unexpectedly increases *P. aeruginosa* lethality in both acute and chronic infections, even while stimulating a stronger immune response by *Caenorhabditis elegans*.

**IMPORTANCE:**

*Pseudomonas aeruginosa*, the ubiquitous environmental bacterium and human pathogen, forms multicellular communities known as biofilms in response to various stimuli. We find that glycerol, a common carbon source that bacteria can use for energy and biosynthesis, encourages biofilm behaviors such as surface attachment and colony wrinkling by *P. aeruginosa*. Glycerol can be derived from surfactants that are present in the human lungs, a common infection site. Glycerol-stimulated biofilm phenotypes do not depend on phosphorylation of glycerol but are surprisingly impacted by a glucose breakdown pathway, suggesting that it is glycerol utilization, and not its mere presence or cellular import, that stimulates biofilm phenotypes. Moreover, the same mutations that block glycerol-stimulated biofilm phenotypes also impact *P. aeruginosa* virulence in both acute and chronic animal models. Notably, a glucose-breakdown mutant (Δ*edd*) counteracts biofilm phenotypes but shows enhanced virulence and stimulates a stronger immune response in *Caenorhabditis elegans*.

## INTRODUCTION

*Pseudomonas aeruginosa* is a ubiquitous Gram-negative environmental bacterium. It is frequently present in human-proximal environments, including in both soil and water (1). An opportunistic pathogen, it causes a variety of infections, ranging from skin and ear infections (2) to pneumonia for which it is rightly notorious (3), particularly in persons living with cystic fibrosis (CF) (4–8). It also commonly infects chronic wounds and burn wounds (9–11). One reason for its characteristic resistance to antimicrobial therapy is its expression of multidrug efflux pumps (12, 13) and acquired resistance genes, such as carbapenemases (14–16). Its treatment tolerance is also attributed to the formation of biofilms (17, 18), in which populations of bacterial cells are surrounded by a protective extracellular matrix consisting of polysaccharides, DNA, and proteins (18, 19). A combination of factors causes antimicrobial tolerance: the biofilm matrix limits the diffusion of drugs (20, 21), cell metabolism is often altered in the stationary phase-like interior to form significant numbers of persister cells (22), and internal hypoxia upregulates efflux pumps (23).

Biofilm communities of *P. aeruginosa* form in response to environmental cues and intercellular signals. For instance, the role of quorum sensing through the *rhl* and *las* acyl homoserine lactone systems has been extensively studied [for a review, see reference (24)]. Nutritional cues are also known to play pleiotropic roles in *Pseudomonas*, as in many other organisms. Deletion of global carbon catabolite repression regulators such as the RNA binding proteins *crc* and *hfq* in *P. aeruginosa* alters the regulation of hundreds of genes involved in biofilm formation, antibiotic resistance, quorum sensing, and virulence (25–28). Glucose increases biofilm thickness in strain PAO1 by affecting quorum sensing (29), while succinate and glutamate enhance the dispersal of established biofilms (30). Carbon source is thus intertwined with behaviors that are important to the foundation and maturation of a biofilm. One study (31) suggested that biofilm formation is reduced in the presence of 20 mM citric acid, though it did not disentangle carbon source identity from the resulting low pH, nor did it assess the production of the biofilm exopolysaccharides Pel or Psl (32–34) by PAO1. Pel is shared by both PAO1 and the highly virulent, clinically prevalent (35) strain PA14 [which does not produce Psl (33)], making Pel an important component of biofilm studies seeking to understand the importance of the polysaccharide matrix in infection.

**Fig 1:**
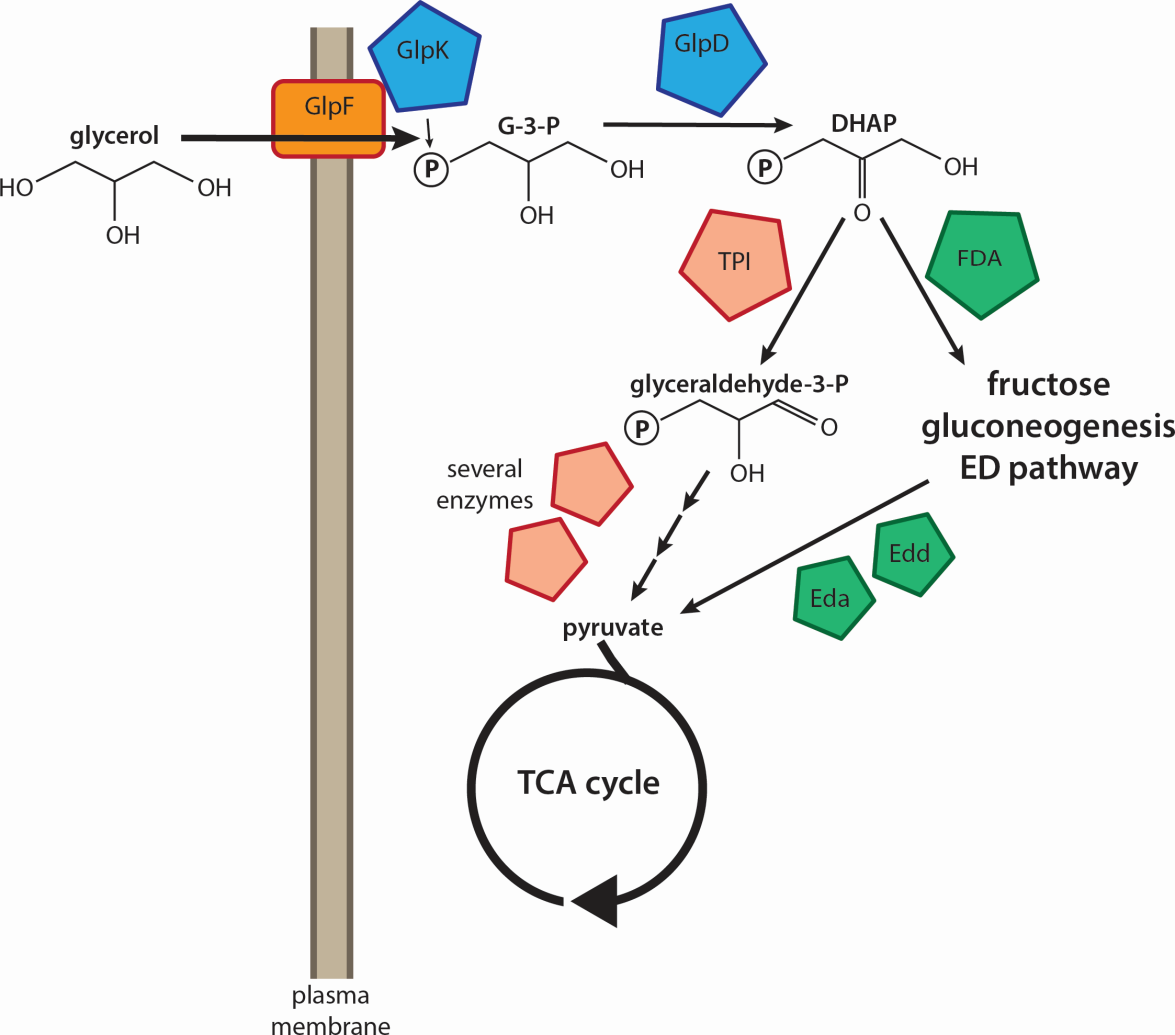
Schematic of presumed glycerol metabolism in *P. aeruginosa*. Glycerol is taken up by diffusion through the facilitator GlpF, which may be associated with the kinase GlpK to immediately phosphorylate incoming glycerol molecules. G-3-P is dehydrogenated by GlpD to DHAP, the central molecule in the pathway. DHAP can be isomerized to glyceraldehyde-3-phosphate by TPI and then converted into pyruvate to feed the TCA cycle (left pathway). Alternatively, DHAP can be converted to fructose by fructose bisphosphate aldolase (FDA). This anabolic production of fructose leads to G-6-P via gluconeogenesis. G-6-P can either be used for biosynthesis or be burned for energy via the ED pathway (right pathway). Within the ED pathway, the Edd and Eda enzymes catalyze the steps in the production of pyruvate from 6-phosphogluconate, which can be derived from G-6-P.

In previous work, we used a screen on M6301 medium supplemented with 0.5% (68 mM) glycerol and 0.2% casamino acids to identify mutations affecting colony wrinkling as a marker for biofilm formation (36). We empirically found that this medium formulation typically made high Pel polysaccharide-expressing strains display a rough, wrinkled colony phenotype, whereas low Pel production corresponded with smooth colonies. This observation was validated by testing the PA14 wild type (WT) against known Δ*amrZ* and Δ*bifA* mutants that produce elevated amounts of Pel (37, 38). In the present study, we investigated whether the use of glycerol as a carbohydrate source encourages biofilm-related behaviors in *P. aeruginosa* PA14. Glycerol can be derived from the common lung surfactant phosphatidylcholine (39), and *P. aeruginosa* growing in the human lung upregulates glycerol catabolism genes (40). It would thus be logical for *P. aeruginosa* to produce a biofilm matrix and establish a persistent infection in the presence of glycerol. Previous studies using *P. aeruginosa* FRD1 and PAO1 showed glycerol-mediated enhancement of surface-attached biofilms but also showed differences between the strains (41), prompting us to study this phenomenon in PA14. Glycerol is metabolized via several intermediates (Fig. 1), with two critical lynchpins thought to be the conversion of glycerol to glycerol-3-phosphate (G-3-P) by the kinase GlpK (42) and the oxidation of G3P to dihydroxyacetone phosphate (DHAP) by the dehydrogenase GlpD (Fig. 1) (43). DHAP can then undergo gluconeogenesis through the fructose bisphosphate aldolase enzyme Fda to produce fructose-1,6-bisphosphate and eventually glucose-6-phosphate (G-6-P) in a cycle that allows the production of polysaccharides and other cell components (44). Alternatively, triose phosphate isomerase (TPI) can convert DHAP into glyceraldehyde-3-phosphate (Fig. 1), which through several subsequent steps enters the TCA cycle as pyruvate (43). The gluconeogenic route, however, is also capable of producing pyruvate (and thus entering the TCA cycle) via the Entner-Doudoroff (ED) pathway, converting G-6-P into the 6-phosphogluconate that is metabolized for energy in this alternative to classical Embden-Meyerhof-Parnas glycolysis, which is not present in *P. aeruginosa* (45). A critical step in this pathway is the conversion of 6-phosphogluconate to 2-keto-3-deoxy-6-phosphogluconate (KDPG), catalyzed by the Edd enzyme (45).

We use a combination of colony morphology examination, biofilm attachment assays, Congo red staining, and gene deletions to examine the role of glycerol in biofilm matrix production. We demonstrate that a glycerol kinase is important but not essential for the growth on glycerol and that the loss of Edd as a key Entner-Doudoroff pathway enzyme that is upregulated during glycerol growth (46) impacts both growth and biofilm phenotypes in glycerol. Finally, we test how two glycerol metabolic pathway mutants compare to wild-type PA14 in their ability to kill *Galleria mellonella* in acute hemolymph infection and *Caenorhabditis elegans* in a gut biofilm infection model.

## RESULTS

### Glycerol stimulates biofilm phenotypes

**Fig 2:**
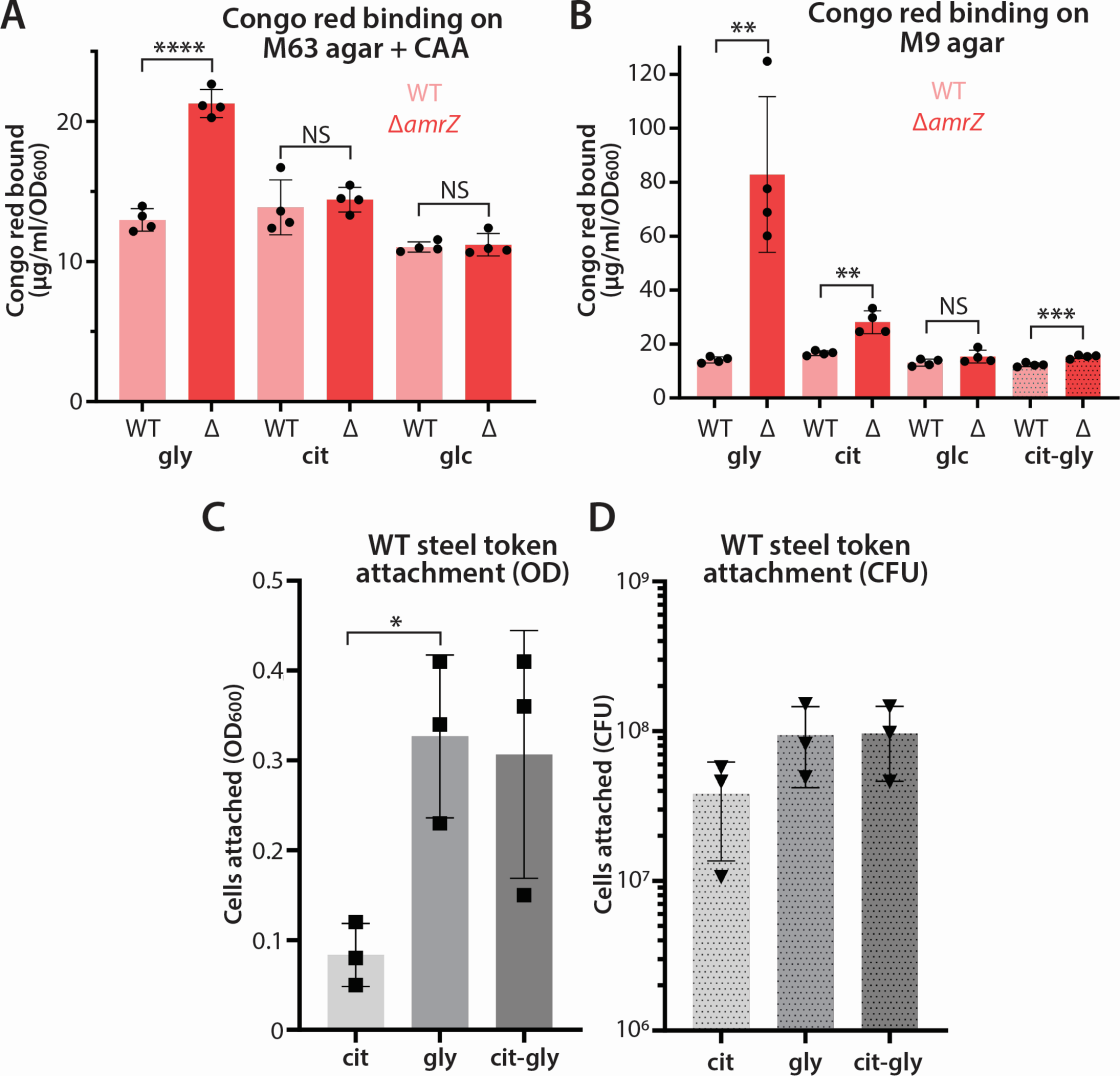
Impact of glycerol on biofilm phenotypes. (**A**) Congo red binding data (micrograms of Congo red bound per milliliter per OD_600_ of cells) for the WT PA14 or Δ*amrZ* strains (Δ) represented as mean values of biological quadruplicate samples (*n* = 4). Strains were grown on solid M63 + 0.2% CAA agar supplemented with 68 mM glycerol, 34 mM citrate, or 34 mM glucose for 6 days at 25°C. (**B**) Congo red binding data as in panel **A** comparing PA14 and Δ*amrZ* on M9 medium with 68 mM glycerol, 34 mM citrate, 34 mM glucose, or a 17 mM citrate + 34 mM glycerol mixture. (**C and D**) Relative number of cells as indicated by OD_600_ (**C**) or serial dilution and colony counting (**D**) in cell suspensions liberated from steel tokens incubated statically with PA14 cells in M9 supplemented with the indicated carbon sources for 4 days at 37°C. Cit, 34 mM citrate; gly, 68 mM glycerol; cit-gly, 17 mM citrate + 34 mM glycerol. In all panels, error bars represent the standard deviation of replicate samples. Statistical pairwise comparisons in panels A and B used an unpaired Student’s *t* test (NS, not significant; **P < 0.01; ***P < 0.001; and ****P < 0.0001). Statistical comparisons in panels C and D used one-way ANOVA followed by Dunnett’s multiple comparisons test, using citrate as the control. *P < 0.05.

We first examined the effect of different carbon sources, including glycerol, on Pel biofilm matrix production by PA14. We sought to test the effects of these carbon sources under different nutritional conditions, including in the M63-glycerol formulation typically used by our lab (36), which also contains casamino acids (CAA) as an available carbon source for *P. aeruginosa* [e.g., references (47–49)]. Using M63 + CAA agar, we inspected Congo red binding, as a measure of Pel extracellular matrix production, by PA14 and the well-studied, moderately hyper-biofilm strain Δ*amrZ*. We observed that addition of 0.5% (68 mM) glycerol, but not of carbon-equivalent (34 mM) citrate or glucose, resulted in a significant elevation in Congo red binding by Δ*amrZ* over the wild type (Fig. 2A). To extend this result, we then assessed Congo red binding by colonies grown on M9-based solid media where the added glycerol, citrate, or glucose was the sole carbon source (medium without added carbohydrate does not support the growth of *P. aeruginosa*; Fig. S1). Under these conditions, we observed the largest difference between PA14 and its Δ*amrZ* derivative on glycerol, while citrate also yielded a significant difference (Fig. 2B). Glucose, however, did not show a significant difference between the two strains (Fig. 2B). We also tested a carbon-equivalent mixture of 17 mM citrate and 34 mM glycerol to learn whether the effects of glycerol and citrate would be additive. Interestingly, we saw less Congo red binding overall under these conditions, but Δ*amrZ* cells retained a significantly greater level of binding than the wild type (Fig. 2B).

As a different measure of biofilm formation, we assessed the ability of PA14 cells to attach to a steel token in a static liquid M9 culture containing carbon-equivalent amounts of citrate, glycerol, or both as the sole carbon source. Here, substantially more cells were liberated from steel tokens when glycerol was the carbon source, as assessed either by the OD_600_ of the resuspended material (Fig. 2C) or by the number of colony-forming units (CFU) in the resuspension (Fig. 2D), supporting the conclusion that glycerol stimulates *P. aeruginosa* surface attachment. This result also suggests that glycerol has a positive effect on biofilm formation, rather than other carbon sources having a negative effect.

### Glycerol highlights differences in colony morphology

**Fig 3:**
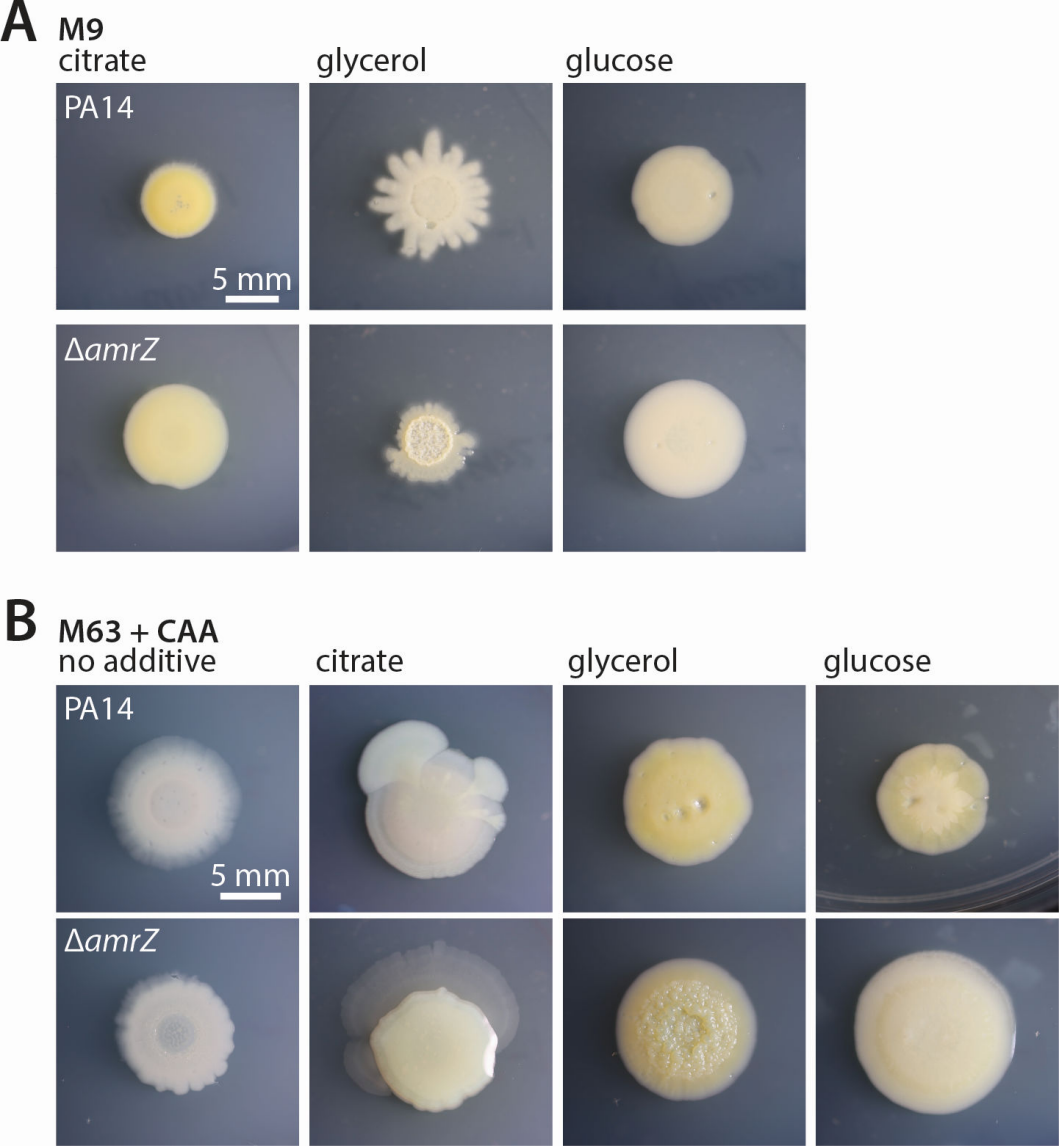
Influence of glycerol on colony appearance. Photographs of representative colonies grown for 6 days at 25°C. (**A**) PA14 or Δ*amrZ* colonies grown on M9-1% agar supplemented with 68 mM glycerol, 34 mM citrate, or 34 mM glucose as indicated. (**B**) PA14 or Δ*amrZ* colonies grown on M63-1% agar + 0.2% CAA agar, either without further supplementation (“no additive”) or with 68 mM glycerol, 34 mM citrate, or 34 mM glucose as indicated.

The effect of glycerol was also noticeable in colony morphology (Fig. 3). Again comparing PA14 cells with their elevated-biofilm Δ*amrZ* derivative, colony wrinkling by the Δ*amrZ* strain was immediately apparent only on glycerol-containing medium, whether as the sole carbon source on M9 medium (Fig. 3A) or in addition to CAA on M63 medium (Fig. 3B). This visual difference is distinct from Congo red-determined Pel levels, as increased Pel (as in the case of Δ*amrZ* on M9-citrate, Fig. 2B) is not necessarily accompanied by a wrinkled colony morphology (Fig. 3A). A visual distinction in colony wrinkling on glycerol-containing medium represents a practical advantage for facile screening of strains that may have altered biofilm phenotypes (36).

### *glpK* is important but not essential for glycerol utilization and modestly impacts biofilm phenotypes on glycerol

The effects of glycerol on biofilm phenotypes led us to ask whether the initial steps in glycerol utilization are important for those phenotypes. The glycerol kinase GlpK binds to the diffusion facilitator GlpF (42), and these two proteins are encoded in an operon. We thus deleted the *glpK* (*PA14_17960*) gene and first examined growth on M9 glycerol to ensure there was a defect; it was previously reported that Δ*glpK* strains do not grow on glycerol (42). The same study (42) also identified a highly similar putative glycerol kinase called GlpK2, whose gene (*PA14_18010*) is divergently transcribed from *glpK*. Hence, we examined the growth and Pel production of individual and double mutants of these two genes.

**Fig 4:**
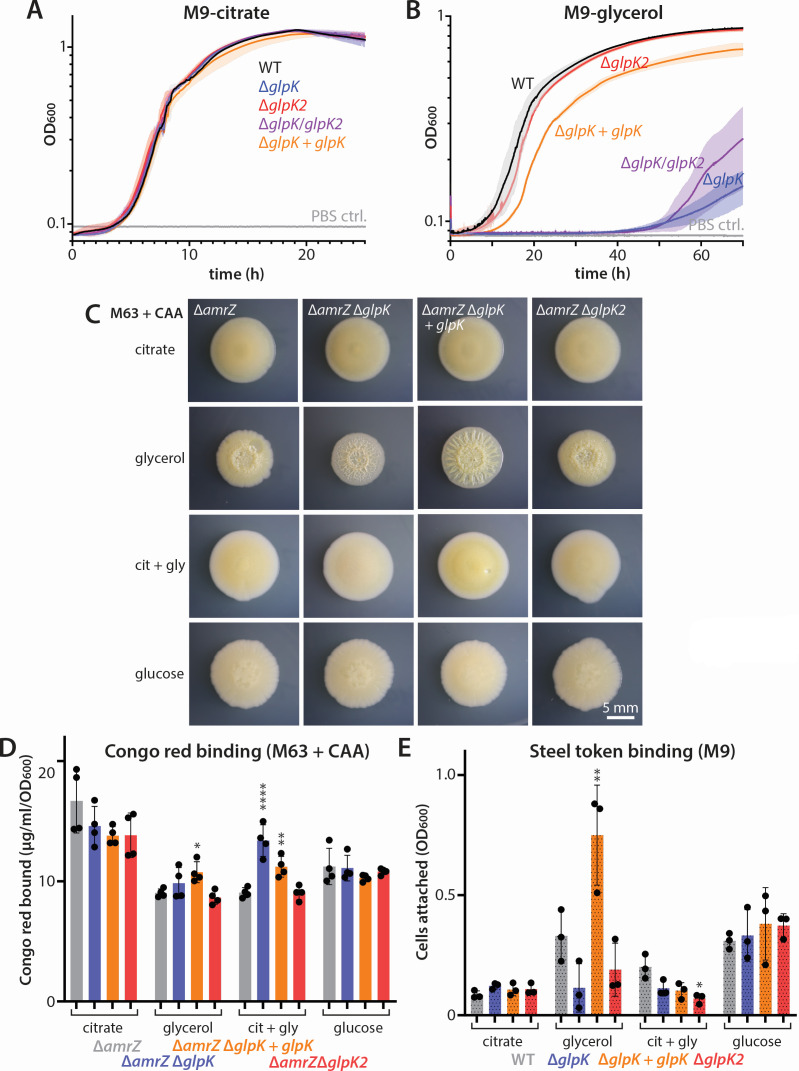
Impact of putative glycerol kinase deletions on glycerol utilization and biofilm phenotypes. (**A**) Growth curves of PA14 (WT) and single or double mutants of suspected glycerol kinases as indicated in M9 supplemented with 34 mM citrate. M9 with sterile phosphate-buffered saline (PBS) added to the medium instead of bacterial culture was used as a contamination control in these long-duration experiments (PBS ctrl). (**B**) Representative growth curves of PA14 (WT) and single or double mutants of suspected glycerol kinases in M9 supplemented with 68 mM glycerol as the sole carbon source. In panels A and B, the curves shown are averages of three biological replicates, each of which is an average of three technical replicates. Shading shows standard deviation among biological replicates. (**C**) Photographs of representative colonies of the indicated strains grown for 6 days at 25°C on M63-1% agar + 0.2% CAA agar, with 68 mM glycerol, 34 mM citrate, 17 mM citrate + 34 mM glycerol, or 34 mM glucose as indicated. (**D**) Congo red binding of the indicated strains grown on the same media as noted in panel C. (**E**) Relative number of cells as indicated by OD_600_ in cell suspensions liberated from steel tokens incubated statically with the indicated strains in M9 supplemented with the indicated carbon sources for 4 days at 37°C. Citrate, 34 mM citrate; glycerol, 68 mM glycerol; cit-gly, 17 mM citrate + 34 mM glycerol; glucose, 34 mM glucose. In panels D and E, error bars represent the standard deviation of replicate samples. The “+ *glpK*” is shorthand for complementation of *glpK* at the *attB* locus. The Δ*amrZ* data in panel C and the wild-type data in panel E are identical to those in Fig. 5C and F, respectively, as all mutants were tested in the same experiments for maximal consistency. Statistical comparisons in panels D and E used one-way ANOVA followed by Dunnett’s multiple comparisons test, using the wild type as the control for each condition. *P ≤ 0.05; **P < 0.01; and ****P < 0.0001.

We found that both the individual and double mutant strains grew normally in M9 supplemented with citrate, as expected (Fig. 4A). In contrast, the Δ*glpK* and Δ*glpK* Δ*glpK2* mutants showed a severe growth defect in M9-glycerol, with long (~50 h) lag in growth (Fig. 4B). This was surprising given the previous report that *glpK* mutants do not grow at all on glycerol; however, that study did not show growth data (42). Interestingly, the Δ*glpK2* single mutant grew identically to the wild type on glycerol, and loss of *glpK2* did not exacerbate the Δ*glpK* phenotype (Fig. 4B). Complementation of the Δ*glpK* strain with *glpK* at an ectopic locus (*attB*) largely restored growth on glycerol (Fig. 4B), indicating that the *glpK* deletion is indeed responsible for the growth delay. These data together suggest that while *glpK2* may encode a highly similar protein, it is not redundant with *glpK*. Moreover, the eventual growth of the Δ*glpK* mutant in a medium with glycerol as the sole carbon source indicates that GlpK is not absolutely required for growth on glycerol and that GlpK-independent pathways for glycerol utilization must exist.

**Fig 5:**
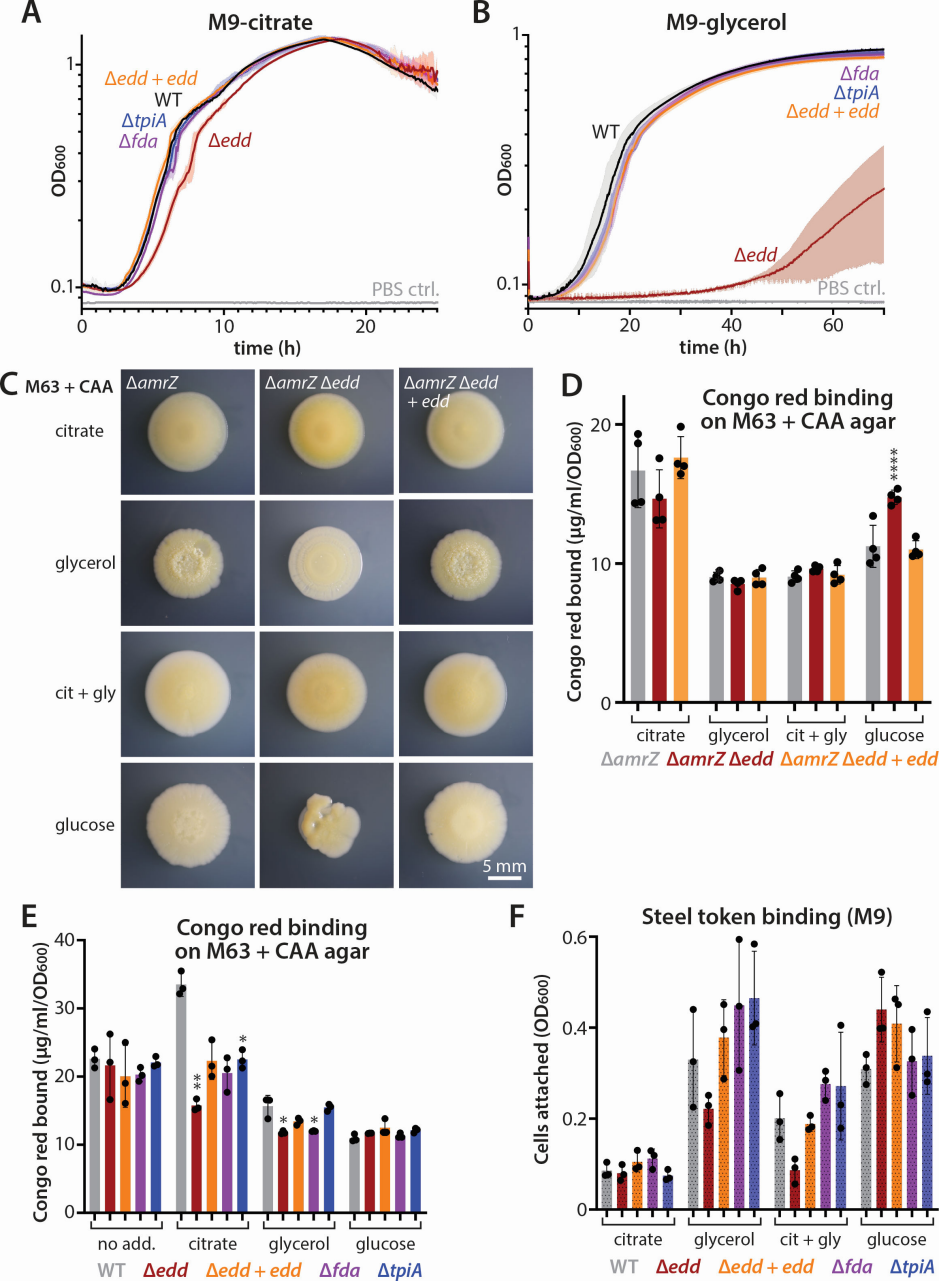
Impact of glycerol metabolism gene deletions on glycerol utilization and biofilm phenotypes. (**A**) Representative growth curves of PA14 (WT) and deletions of the indicated genes in M9 supplemented with 34 mM citrate as the sole carbon source. (**B**) Representative growth curves of the same strains as in panel A but with 68 mM glycerol as the sole carbon source. M9 with sterile phosphate-buffered saline (PBS) added to the medium instead of bacterial culture was used as a contamination control in these long-duration experiments (PBS ctrl). The curves shown are averages of three biological replicates, each of which is an average of three technical replicates. Shading shows standard deviation among biological replicates. The “+ *edd*” is shorthand for complementation of *edd* at the *attB* locus. (**C**) Photographs of representative colonies of the indicated strains grown for 6 days at 25°C on M63-1% agar + 0.2% CAA agar, with 68 mM glycerol, 34 mM citrate, 17 mM citrate + 34 mM glycerol, or 34 mM glucose as indicated. (**D**) Congo red binding of the indicated strains grown on the same media as noted in panel C. (**E**) Congo red binding of the indicated strains, here in a wild-type (not Δ*amrZ*) background on the same media as noted in panel C, except that cit + gly is replaced with medium containing no added carbon source (“no add.”). (**F**) Relative number of cells as indicated by OD_600_ in cell suspensions liberated from steel tokens incubated statically with the indicated strains in M9 supplemented with the indicated carbon sources for 4 days at 37°C. Citrate, 34 mM citrate; glycerol, 68 mM glycerol; cit-gly, 17 mM citrate + 34 mM glycerol; and glucose, 34 mM glucose. Error bars in panels D–F show the standard deviation of the biological replicates. The “+ *edd*” is shorthand for complementation of *edd* at the *attB* locus. The Δ*amrZ* images in panel C and data in panel D are identical to those in Fig. 4C and D; the wild-type data in panel E are identical to those in Fig. S3B; and the wild-type data in panel F are identical to those in Fig. 4E, as all mutants were tested in the same experiment for maximal consistency. Statistical comparisons in panels D–F used one-way ANOVA followed by Dunnett’s multiple comparisons test, using the wild type (or Δ*amrZ*) as the control for each condition. *P ≤ 0.05; **P < 0.01; and ****P < 0.0001.

We next assessed the impact of *glpK* and *glpK2* deletions on biofilm formation, both in the wild-type background and in Δ*amrZ* strains, where changes in colony morphology might be more apparent. Because Δ*glpK* cells grow poorly on glycerol alone, we looked at colony morphology and Congo red binding on M63 + CAA agar with different carbon sources. In the Δ*amrZ* background, wrinkled colony morphology was only apparent on glycerol, not on citrate, glucose, or a mixture of citrate and glycerol (Fig. 4C). On glycerol, deletion of *glpK* changed the appearance of the colony, eliciting a fine, reticulated pattern of wrinkles (Fig. 4C). Complementation of the *glpK* deletion yielded colonies with a clear, radially spoked morphology that was distinct from the Δ*amrZ* parent (Fig. 4C). However, deletion of *glpK2* did not discernably change the colony structure. Estimation of Pel levels using Congo red binding revealed that wrinkling does not correspond to greater Pel production; in fact, the featureless citrate colonies bound more Congo red than the glycerol-grown colonies (Fig. 4D). Similarly, we saw significant differences in Congo red binding among the strains grown on the citrate-glycerol mixture, but none of the strains exhibited colony wrinkling (Fig. 4C and D). On both glycerol and the citrate-glycerol mixture, the *glpK*-complemented strain showed modest but significant increases in Pel (Fig. 4D), a result that is at least concordant with its more salient wrinkling. We observed similar results in the wild-type background, but with less-pronounced colony wrinkling (Fig. S3A), and the same disconnect between cell density-corrected Pel levels and colony morphology; colonies grown on citrate or even without an added carbon source bound at least as much Congo red as glycerol-grown colonies (Fig. S3B). Surprisingly, deletion of either *glpK* or *glpK2* lowered Congo red binding on citrate-amended medium, with Δ*glpK2* reaching statistical significance (Fig. S3B). While this effect remains unexplained at present, it is unlikely to be related to glycerol metabolism, given that it occurred on citrate and also with *glpK2* deletion, which had no effect on glycerol utilization (Fig. 4A). We also assessed biofilm formation using the steel token-binding assay, using a mixture of citrate and glycerol as a way to circumvent the poor growth of the Δ*glpK* strain on glycerol alone (Fig. S2). Unlike on citrate or glucose, *glpK* and *glpK2* deletion strains tended to show less attachment to the steel token in glycerol-containing medium, with only Δ*glpK2* reaching statistical significance (Fig. 4E). Complementation of *glpK* significantly increased token binding in glycerol (Fig. 4E), consistent with the restored growth (Fig. 4B) and enhanced colony wrinkling (Fig. 4C) of this strain. However, both the *glpK*-complemented strain and the Δ*glpK2* strain showed lower token binding in citrate + glycerol medium (Fig. 4E). Collectively, these data suggest that GlpK, while important for growth on glycerol, has only a modest impact on biofilm phenotypes.

### Blockage of Entner-Doudoroff glucose catabolism impacts glycerol utilization and biofilm formation

To test whether glycerol metabolism downstream of phosphorylation is required for biofilm stimulation by glycerol, we perturbed the other end of the glycerol metabolism pathway, namely energy production and anabolism. We deleted *fda* (*PA14_07230*), *tpiA* (*PA14_62830*), or *edd* (*PA14_22910*), each of which encodes a key enzyme in glycerol metabolism: anabolic production of fructose/glucose, direct breakdown of DHAP and use in the TCA cycle, and the derivation of energy from anabolically produced glucose, respectively. We first looked for glycerol growth defects, as might be expected from a gluconeogenic deficit. On citrate, all the strains grew well, with Δ*edd* showing a very minor growth defect that was corrected by complementation (Fig. 5A). Unexpectedly, we found that Δ*fda* and Δ*tpiA* did not grow significantly differently from PA14 on glycerol (Fig. 5B). However, Δ*edd* exhibited a very long lag (about 50 h, compared to under 10 h for the other strains) and poor growth rate compared to PA14, an effect that was fully reversed by ectopic complementation with *edd* (Fig. 5B). This observation matches previous data showing elevated Edd enzyme activity (50) and ED pathway transcription (46) in glycerol-grown cells (compared to TCA cycle intermediate-grown cells) without any obvious induction of *tpiA* or *fda* transcription (46), though these enzymes are likely still active.

Given the glycerol growth defect of Δ*edd* (and lack thereof in the other two mutants), we assayed biofilm formation on M63 + CAA either unamended or supplemented with citrate, glycerol, or glucose. We again used the Δ*amrZ* strain background to visualize colony morphology in Δ*edd* mutants, while also testing all the mutants in a wild-type background. In the Δ*amrZ* background, colony wrinkling was again only visible on glycerol-containing medium (Fig. 5C). Notably, deletion of *edd* abrogated wrinkling, while *edd* complementation restored it (Fig. 5C). However, these differences in wrinkling did not correspond to Congo red binding differences (Fig. 5D). Interestingly, deletion of *edd* did prompt a significant increase in Pel levels in colonies grown on glucose, a phenomenon accompanied by a change in colony shape but not wrinkling (Fig. 5C and D). Our observations in the wild-type background agreed with the Δ*amrZ* results. On unamended medium and glucose-supplemented medium, none of the deletions significantly impacted colony morphology (Fig. S4A) or Congo red binding (Fig. 5E). On glycerol-supplemented medium, both the Δ*edd* and Δ*fda* mutants exhibited modest but statistically significant decreases in Congo red binding, an effect that was at least partially rescued by *edd* complementation (Fig. 5E). As with the *glp* deletions, all of the deletions, and even the *edd*-complemented strain, showed decreased Congo red binding relative to the wild type on citrate, with Δ*edd* and Δ*tpiA* reaching statistical significance (Fig. 5E). We have no satisfactory explanation for this effect; if we had uncovered a true function of these genes in Pel production by citrate-grown cells, we would have expected full rescue in the *edd-*complemented strain. Moreover, on M9-citrate agar, none of the mutants bound Congo red significantly differently from the wild type (Fig. S4B), suggesting that the phenotype at least is not citrate mediated.

We also examined biofilm formation on steel tokens. We observed that none of the deletions impacted token binding in citrate, but that Δ*edd* had a (non-statistically significant) binding defect in glycerol and citrate-glycerol that was reversed by *edd* complementation (Fig. 5F). Interestingly, in glycerol-containing medium, the *fda* and *tpiA* deletions both tended toward higher biofilm formation, but this effect did not reach statistical significance (Fig. 5F). Overall, among the ED pathway mutants we tested, deletion of *edd* stood out as negatively impacting both growth in glycerol and biofilm formation, especially in glycerol-containing media.

### Glycerol metabolic mutations impact virulence in acute and chronic infection models

Because we saw impacts on biofilm formation Pel levels in the tested deletions, particularly Δ*edd*, we evaluated *in vivo* whether these strains have different virulence properties in an acute hemolymph infection model (*Galleria mellonella*) and a chronic biofilm infection model (*Caenorhabtidis elegans* gut). We initially predicted that there would be no differences in *G. mellonella* among the strains, whereas in *C. elegans,* the biofilm deficiencies of Δ*glpK* and Δ*edd* might result in hypovirulence.

**Fig 6:**
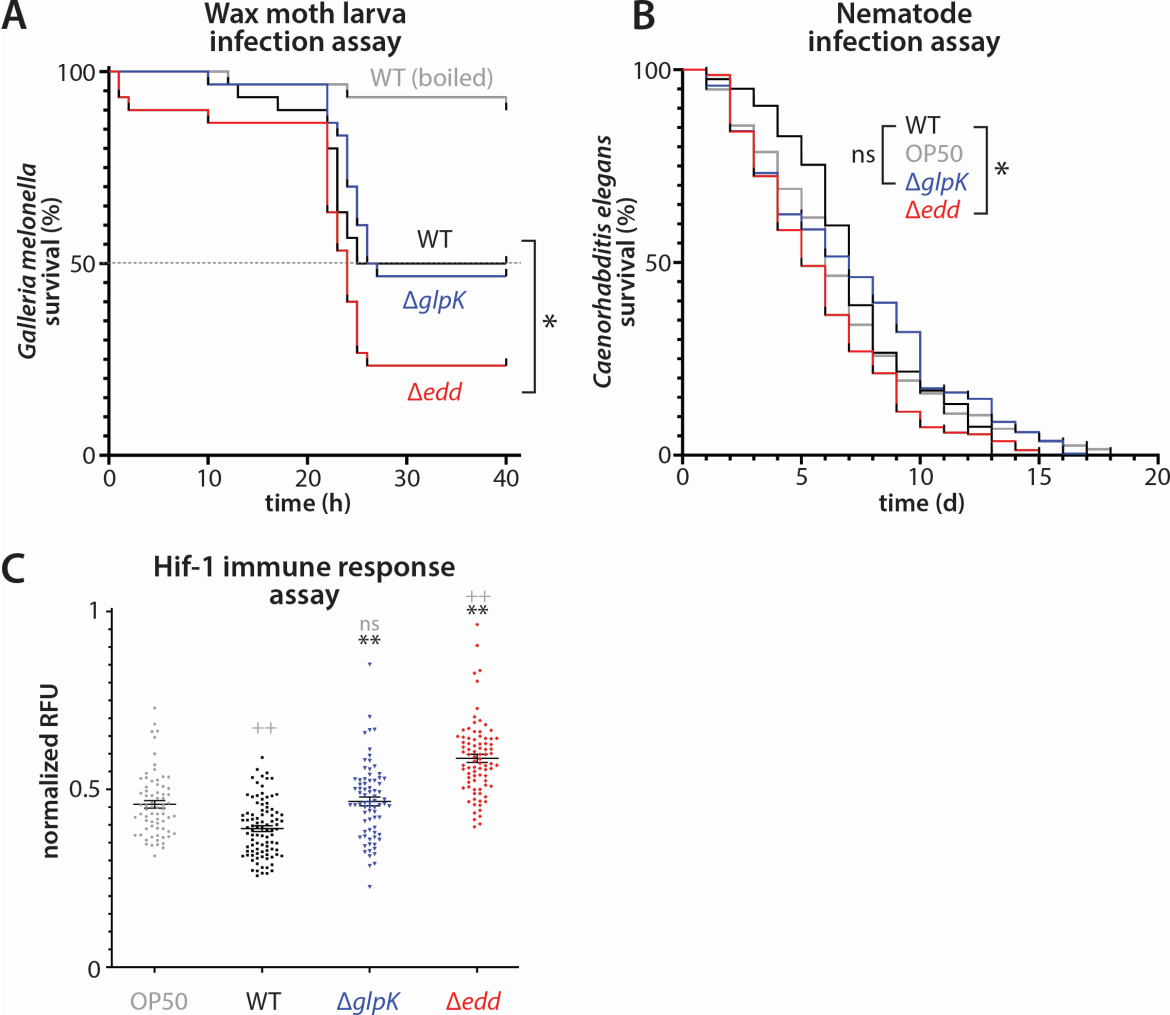
Impact of metabolic mutants in acute and chronic invertebrate infection models. (**A**) Survival curves of wax moth larvae (*Galleria mellonella*) subjected to acute hemolymph infection with PA14, Δ*glpK*, or Δ*edd* strains as indicated. Heat-killed PA14 was used as a control. Asterisk indicates significantly different survival vs PA14. (**B**) Survival curves of nematodes (*Caenorhabditis elegans*) subjected to gut biofilm infection with the indicated strains. *Escherichia coli* OP50 was used as a non-infecting control. Graphs represent combined experimental results for animals fed on the indicated strains prior to shifting. Days are reported post-infection. Asterisk indicates significantly different survival vs PA14 (*P* < 0.05). Data in panels A and B are presented as Kaplan-Meier survival curves. Statistical significance was estimated by the Mantel-Cox log-rank test. (**C**) Representative plots of immune responses in *C. elegans* animals infected with the indicated strains as assessed by fluorescence intensity of a HIF-1-responsive reporter. The mean and SEM are shown with the individual values. Significance in gray is vs OP50. ++*P* < 0.0001 and ns, not significant. Significance in black is vs PA14. **P < 0.0001.

However, contrary to our expectations, the Δ*edd* strain caused higher mortality (about 73%) than either PA14 (50%) or Δ*glpK* (about 57%) in the *G. mellonella* infection model (Fig. 6A). The Mantel-Cox log-rank test confirmed that Δ*edd* is significantly more lethal than PA14 (*P* < 0.05). All three tested strains caused significantly elevated levels of mortality compared to the boiled-PA14 negative control (10% mortality). We presently have no satisfying explanation for why *edd* deletion increases virulence. Nonetheless, and consonant with our initial hypothesis, neither of the deletions resulted in hypovirulence compared to PA14.

In the *C. elegans* infection model, unlike *G. mellonella* infection, the experiment runs until even the *Escherichia coli* OP50-fed control dies. In this case, we examined the median lifespan and time to population extermination to determine differences. The Mantel-Cox log-rank test was again used to estimate statistical significance based on these two parameters. We found that worms infected with biofilm mutants were totally exterminated at a slightly later time compared to PA14 (13 days for PA14; 15 days for Δ*edd*; and 17 days for Δ*glpK* compared to 18 days for the OP50 negative control) (Fig. 6B). However, the median lifespan of the animals infected with Δ*edd* (5 days) was shorter than for PA14 or Δ*glpK* (both 7 days), as the nematodes began to die more slowly when fed on PA14 than when fed on either mutant (or even *E. coli* OP50). Taking the entire killing curve into consideration, only Δ*edd* was considered significantly different from PA14. While Δ*glpK* was neither significantly different from PA14 nor from OP50, its killing curve was distinct in shape from PA14 (Fig. 6B), rendering the overall effect of *glpK* loss on virulence in this model inconclusive. Unexpectedly, but consistent with the data from the acute larval infection model (Fig. 6A), Δ*edd* appeared to be more virulent than the wild type.

### Glycerol metabolic mutations affect the immune response of infected *C. elegans*

Intrigued by the apparent hypervirulence of the Δ*edd* strain, but not the Δ*glpK* strain, we examined *P. aeruginosa*-fed *C. elegans* bearing a HIF-1 responsive GFP reporter to monitor the immune response of infected nematodes. We found that while wild-type PA14 slightly but significantly repressed the nematodes’ immune response relative to the *E. coli* OP50 control (Fig. 6C), this repression was not present for the Δ*glpK* mutant, implying that impaired glycerol metabolism may somehow affect the *C. elegans* immune response to *P. aeruginosa*. More strikingly, the Δ*edd* mutant caused a significantly elevated immune response relative to the OP50 control (Fig. 6C). This elevated immune response is at least consistent with the hypervirulence exhibited by Δ*edd* in both infection models we tested.

## DISCUSSION

One intriguing effect of glycerol, relative to other growth-supporting carbohydrates, is its ability to magnify differences in colony morphology among strains with different genotypes (Fig. 2A and B, Fig. 3, 4C, and 5C). For example, glycerol uncovered differences between wild-type and Δ*amrZ* strains that were not apparent on other carbon sources (e.g., citrate or glucose). This property of growth on glycerol helps to explain why a glycerol-based growth medium was well-suited to visually screen mutants for biofilm phenotypes (36). Importantly, glycerol need not be the sole carbon source of *P. aeruginosa* to stimulate biofilm formation, as the addition of glycerol to casamino acid-supplemented media also resulted in the ability to discern variations in colony wrinkling. While in some cases, increases in colony wrinkling are accompanied by an increase in Pel levels as assessed by Congo red binding (Fig. 2A and B), this is in no way a strict correlation. On the contrary, we observed that citrate-grown colonies, which were largely featureless, typically bound more Congo red than glycerol-grown colonies (Fig. 4D and 5E). These findings suggest that colony wrinkling alone is an unreliable indicator of exopolysaccharide levels. Interestingly, we did observe a weak correlation between the ability of a particular strain in a particular carbon source to bind to a steel token and the appearance of wrinkled colony morphology. For instance, we saw enhanced wrinkling of the Δ*glpK + glpK*-complemented strain in an Δ*amrZ* background and enhanced token binding by the same strain in the wild-type background (Fig. 4C and E). Similarly, colony wrinkling was abrogated in the Δ*amrZ* Δ*edd* strain, and *edd* deletion also lowered token binding in the wild-type background (Fig. 5C and F). Hence, we speculate that colony wrinkling may be related to cell adhesion, perhaps to other cells and/or the agar surface.

Deletion of *glpK* or *edd*, besides hampering growth on glycerol, also impacted colony morphology and steel token binding. How does glycerol uptake and utilization connect to biofilm phenotypes? Loss of the glycerol kinase GlpK, but not its paralog GlpK2, led to a marked growth defect on glycerol, implying that GlpK plays a primary role in glycerol utilization and that GlpK2 is unable to compensate for loss of GlpK. However, even Δ*glpK* and Δ*glpK*Δ*glpK2* strains eventually showed some growth on glycerol as the sole carbon source (Fig. 4B), highlighting the existence of a glycerol utilization pathway that either uses an alternative kinase or bypasses glycerol phosphorylation. Obvious alternatives present in other bacteria, such as the DhaK pathway that oxidizes glycerol to dihydroxyacetone before phosphorylating it (51, 52), appear to be absent from *P. aeruginosa* PA14 (53). Hence, uncovering the pathway that is active in Δ*glpK* strains warrants further study. GlpK-deficient cells did not show decreased Pel levels in colony biofilms (Fig. 4D; Fig. S3B) but did show modest decreases in steel token attachment (Fig. 4E), suggesting that glycerol phosphorylation may have a role in cell attachment but not in exopolysaccharide production.

We dissected the putative glycerol anabolic and catabolic pathways to determine which are required for biofilm promotion. Loss of *fda* should interfere with or cut off gluconeogenesis, *tpiA* deletion should block direct shunting of glycerol into the TCA cycle, and *edd* loss should impede the catabolism of anabolically produced glucose. We predicted that interfering with gluconeogenesis by deleting *fda* might abolish cells’ ability to generate the oligosaccharides needed to make a biofilm matrix. The Δ*fda* cells grew normally on glycerol and, while they showed slightly but significantly decreased Pel levels (Fig. 5E), they had no defect in steel token binding (Fig. 5F). A Δ*tpiA* mutant, which we expected to have perhaps slower growth but no biofilm defect, behaved like the wild type. These results suggest an unappreciated backup method of generating hexoses in *P. aeruginosa*, as cells cannot survive without generating saccharides for cell wall synthesis and other anabolic processes. Because we suspected that Δ*tpiA* cells gained energy via catabolism of gluconeogenically produced sugars, we interrupted the ED pathway while maintaining an intact *tpiA*. The Δ*edd* strain cannot convert 6-phosphogluconate to KDPG, a critical step in the catabolism of glucose in *P. aeruginosa* (45). Because cells in principle can still generate energy via the TPI pathway and saccharides through Fda, we did not expect to see a phenotype in this strain. Surprisingly, we found that this mutant had the strongest growth and biofilm phenotype among the metabolic pathway mutants we tested (Fig. 5), implicating glucose metabolism via the ED pathway as critical to glycerol metabolism and hence to biofilm formation in *P. aeruginosa*. We are thus tempted to speculate that interruption of the ED pathway may represent a successful biofilm-busting drug design strategy, offering many potential target enzymes that are not present in humans (unlike targets such as glycerol kinase).

Given their different biofilm defects and altered metabolism, we hypothesized that Δ*glpK* and Δ*edd* strains might display altered virulence in a biofilm infection setting. We thus compared acute infection (injection of *P. aeruginosa* into *G. mellonella* hemolymph) to a gut biofilm lifestyle (temporary feeding of *P. aeruginosa* to *C. elegans*), expecting that our Δ*edd* and Δ*glpK* mutants might act like PA14 in the *G. mellonella* model while possibly displaying reduced virulence in *C. elegans* due to exopolysaccharide or surface-binding deficiencies. We found that Δ*edd* was in fact slightly more lethal in *G. mellonella* (Fig. 6A)*,* which, while surprising, did not run counter to our hypothesis. In *C. elegans*, the Δ*edd* strain surprisingly showed enhanced virulence, with faster median animal death (Fig. 6B). Moreover, unlike wild-type cells, which suppress the *C. elegans* HIF-1-mediated immune response, both mutants stimulated the immune response, with Δ*edd* causing the strongest response (Fig. 6C). As *P. aeruginosa* typically suppresses HIF-1 via quinolones such as the *Pseudomonas* quinolone signal (PQS) (54), one possible mechanism for the enhanced immune stimulation by Δ*edd* and Δ*glpK* strains is reduced PQS production. Neither of our infection assays yielded evidence that *glpK* deletion strongly affects virulence, yet the differences in the killing curves in both models between wild-type and Δ*glpK* cells (Fig. 6A and B), as well as the difference in the HIF-1 response when infected with Δ*glpK* (Fig. 6C), make us unable to state conclusively that *glpK* deletion does not impact virulence. Interestingly, both *glpK* and *edd* appear to impact *P. aeruginosa* survival in infection settings as assessed by Tn-seq analysis. In authentic CF sputum-containing but otherwise defined medium, an essential role for *edd* (as well as for *fda* and *tpiA*) was apparent, but this was not the case for *glpK* (55), suggesting that the nutritional conditions in CF sputum require utilization of DHAP and the ED pathway but not glycerol phosphorylation. Meanwhile, in acute mouse burn wounds, inactivation of *glpK*, *fda*, or *tpiA* had no effect, while inactivation of *edd* benefitted *P. aeruginosa* survival (11). In a mouse chronic surgical wound model, however, both *glpK* and *edd* were important for survival (11). A third Tn-seq study (56) also found *edd* to be important for biofilm growth in PA14 and a clinical isolate, but not in PAO1, again highlighting strain-specific differences. Finally, deletion of *edd* had no effect in a murine catheter-associated urinary tract infection model (57), suggesting that the infection niche is also important. Together, these data suggest (i) that whether Edd is a liability or asset depends on the type of infection and (ii) that glycerol may be an important nutrient source in chronic infections. Consistent with *edd* inactivation aiding survival in an acute burn wound model (11), our Δ*edd* strain showed more hypervirulence in the acute *Galleria* infection assay than in the more-chronic nematode assay (Fig. 6A and B).

The use of glycerol by *P. aeruginosa* has been previously linked to virulence factor production (58) and biofilm attachment as measured by crystal violet (41). Liberation of glycerol from the lung surfactant phosphatidylcholine is possible (39), and *P. aeruginosa* cells growing in the lung appear to upregulate glycerol utilization genes (40). Together, these data suggest that glycerol may be an important growth substrate and developmental cue for *P. aeruginosa* during host colonization.

In sum, we show that glycerol uniquely brings out colony morphology in *P. aeruginosa* PA14 and also stimulates surface binding. This may not be true in the less-virulent PAO1 strain, whose biofilm development process can be stimulated by glucose (59), perhaps through the Psl system that PA14 lacks. It will be interesting in the future to learn whether Pel in PAO1 is as affected by glycerol as it is in PA14. Because PA14 has only one biofilm matrix polysaccharide, it represents a facile model to understand its regulation without confounding factors. We also identify the Entner-Doudoroff pathway, a key feature of *Pseudomonas* glucose catabolism, as a candidate pathway for therapeutic inhibition of glycerol-stimulated biofilm infections. It will be important in the future to determine how ED pathway blockage by *edd* deletion interdicts glucose utilization, as glycerol utilization represents a secondary target area that may also lead to new antimicrobial therapies for chronic *Pseudomonas* affliction.

## MATERIALS AND METHODS

### Bacterial strains and growth conditions

*E. coli* and *P. aeruginosa* (Table 1) were grown in LB (Lennox) medium (10 g/L tryptone, 5 g/L yeast extract, and 5 g/L NaCl) for overnight cultures. All liquid cultures were grown shaking in 14-mL round-bottom tubes at 180 RPM and 37°C unless otherwise specified. M9 medium was made according to Cold Spring Harbor protocols (60) without glucose, and carbohydrates were added at 34 mM for glucose, 34 mM for citrate, and 0.5%, vol/vol (68 mM) for glycerol to obtain the molar equivalence of carbon atoms. Mixtures of glycerol and citrate were 34 mM glycerol and 17 mM citrate to maintain carbon equivalence. In all cases, cells were pelleted and washed twice with PBS pH 7.4 in order to remove any residual carbohydrates from overnight growth before experiments were performed. M63 was formulated as previously reported (36) with 0.2% casamino acids. Strains bearing pEXG2 were grown in an additional 20 µg/mL gentamycin.

**TABLE 1:** *P. aeruginosa* strains used in this study^*a*^

Strain number	Strain	Characteristics	Source or reference
	*P. aeruginosa* strains		
MTC1	PA14	Laboratory wild type of *P. aeruginosa* PA14	(36)
MTC590	Δ*amrZ*	PA14 with markerless *amrZ* gene deletion	(36)
MTC1210	*attTn7*::*miniTn7T-Gm-GW*::P*_cdrA_-gfp*	PA14 with *attTn7*-integrated c-di-GMP-responsive P*_cdrA_*-gfp reporter	This study
MTC2682	Δ*glpK* (*PA14_17960*)	PA14 with markerless *glpK* deletion	This study
MTC2683	Δ*glpK* Δ*glpK2*	PA14 with markerless *glpK* and *glpK2* deletions	This study
MTC2684	Δ*glpK2* (*PA14_18010*)	PA14 with markerless *glpK2* deletion	This study
MTC2685	Δ*fda* (*PA14_07230*)	PA14 with markerless *fda* deletion	This study
MTC2686	Δ*tpiA* (*PA14_62830*)	PA14 with markerless *tpiA* deletion	This study
MTC2687	Δ*edd* (*PA14_22910*)	PA14 with markerless *edd* deletion	This study
MTC2783	Δ*edd attB*::*edd*	PA14 with markerless *edd* deletion and complementation of *edd* under its native promoter in CTX-1 at *attB*	This study
MTC2784	Δ*glpK attB*::*glpK*	PA14 with markerless *glpK* deletion and complementation of *glpK* under its native promoter in CTX-1 at *attB*	This study
MTC2789	Δ*amrZ* Δ*glpK*	PA14 with markerless *amrZ* and *glpK* deletions	This study
MTC2790	Δ*amrZ* Δ*glpK attB*::*glpK*	PA14 with markerless *amrZ* and *glpK* deletions and complementation of *glpK* under its native promoter in CTX-1 at *attB*	This study
MTC2791	Δ*amrZ* Δ*glpK2*	PA14 with markerless *amrZ* and *glpK2* deletions	This study
MTC2792	Δ*amrZ* Δ*edd*	PA14 with markerless *amrZ* and *edd* deletions	This study
MTC2793	Δ*amrZ* Δ*edd attB*::*edd*	PA14 with markerless *amrZ* and *edd* deletions and complementation of *edd* under its native promoter in CTX-1 at *attB*	This study

^*a*^ Tables with plasmids and *E. coli* strains used are given in the supplemental material.

### Strain construction

*P. aeruginosa* allelic replacement mutants were generated using the pEXG2 plasmid (61) containing the flanking homologous regions of the gene to be deleted, which were made by standard PCR. Plasmids were mobilized into *P. aeruginosa* by conjugation with *E. coli* strain SM10 on LB agar (see supplemental material for SM10 strain list).

### Congo red binding experiments

Cells were washed twice in PBS, and the OD_600_ was measured at 10-fold dilution before dilution to a starting OD_600_ of 0.1, of which 2 µL was spotted onto solid M9 or M63 medium (1% agar) supplemented with the noted carbohydrate source. Plates were then incubated right side up at approximately 25°C (room temperature of the laboratory) for 6 days. After 6 days, each colony was scraped off the agar with a spatula and disrupted in 1 mL of PBS (pH 7.4) in a 1.5-mL centrifuge tube using a Cole-Parmer motorized pestle mixer (catalog number 44468-25). Any Pel flakes were allowed to settle in the PBS, and 100 µL of liquid was removed in order to read the OD_600_ in a 96-well plate in a BioTek Synergy H1 (BioTek, USA) plate reader at twofold dilution (100 µL PBS added to dilute). The remaining sample was centrifuged at 14,000 *g* for 4 min. PBS was removed and replaced with 40 µg/mL Congo red in PBS, and the sample was resuspended by pipetting. Samples were then placed on an orbital shaker and allowed to shake gently. After 1.5 h, samples were centrifuged at 14,000 *g* for 4 min, and 200 µL of the supernatant was pipetted into a 96-well plate. The OD_490_ of the samples was read along with the OD_490_ of a set of standards (40, 20, 10, 5, 2, 1, and 0.5 µg/mL Congo red). The amount of Congo red left in the solution was calculated using this standard curve, subtracted from the starting amount, and the remainder (the amount bound by the biofilm) was divided by the OD_600_ in order to account for differences in colony size. The average and standard error in the mean of biological triplicate results were recorded and plotted as bar charts. Statistical comparisons used one-way ANOVA followed by Dunnett’s multiple comparisons test, using the wild type (or Δ*amrZ*) as the control for each condition.

### Steel token experiments

To study biofilm attachment to a surface, PA14 cells grown overnight in LB were washed twice with PBS and diluted to a starting OD_600_ of 1.0, then diluted 100-fold into 3.5 mL of M9 with either 34 mM citrate, 68 mM glycerol, or 17 mM citrate + 34 mM glycerol in a 12-well polystyrene plate (Corning). An autoclaved steel token was placed in the well, and the plate was incubated statically for 4 days. On the fourth day, the token was removed with sterile forceps, placed in 4 mL of fresh PBS in a glass tube with 0.5 g of 0.5 mm glass spheres, and vortexed for 15 seconds; the OD_600_ of the resulting cell suspension was then measured and/or serially diluted for CFU enumeration. Statistical comparisons used one-way ANOVA followed by Dunnett’s multiple comparisons test, using the wild type as the control for each condition.

### Colony photography

After 6 days of culture at 25°C on M9 or M63-1% agar (same conditions as for Congo red), colonies were photographed using a Canon EOS Rebel T7i camera equipped with a Canon Macro Lens EF-S 35 mm lens. Photographs were taken under soft white lighting on a black-felted copy stand.

### Growth curve experiments

Overnight cultures were washed twice in PBS and diluted 100-fold into a 96-well plate containing appropriate growth medium. The plate was then incubated at 37°C in a Biotek Synergy H1 plate reader with a shake + OD_600_ read step every 5 min.

### *Galleria mellonella* infection experiments

Cultures of *P. aeruginosa* strains were washed twice in PBS and diluted to a concentration of 5 CFU/10 µL. *G. mellonella* larvae were cooled to 4°C for several hours to render them inactive. Worms were then removed from their bedding, and their abdominal region was wiped with 70% ethanol and spotted with 0.5 µL of 25 µg/mL chloramphenicol at the injection site. Five CFU of bacteria were then loaded into a Hamilton syringe equipped with a 22-gauge needle and injected into the worm. The larvae were then incubated at 37°C and observed every hour. Worms that did not respond to gentle prodding using forceps were scored as dead and removed from the experiment.

### *Caenorhabditis elegans* shifting assay

Wild-type Bristol (N2) and UL1447: *leEx*1447 [*hif-1*::GFP +*unc-119*(+)] *Caenorhabditis elegans* were used in this study (*Caenorhabditis* Genetics Center, MN, USA). *P. aeruginosa* and *Escherichia coli* OP50 lawns were created on 100-mm plates using 500 µL of a 15.25 mg/mL (based on cell pellet mass) suspension diluted from an overnight culture, incubated at 37°C for 1 h, then returned to room temperature prior to the addition of *C. elegans. C. elegans* were synchronized using a bleaching protocol and grown until the L4 stage at 20°C (62). The worms were transferred onto the *P. aeruginosa* lawns to feed for 24 h at 20°C. Following the 24-h infection feeding, the worms were gravity washed briefly in M9 buffer containing 200 µg/mL neomycin to kill any *P. aeruginosa* cells that were not ingested (63) and again gravity washed three times in M9 to remove any residual neomycin before loading the worms into the microfluidic chips (Infinity Chips; NemaLife, Inc., TX, USA) (64, 65). Before use, the interiors of the microfluidic chips were prepared according to the manufacturer’s protocol. The worms in the chips were flushed and fed daily with 15.25 mg/mL OP50. The chips were incubated in humidified chambers at 20°C until all animals perished (66). Videos were acquired each day after flushing and before feeding fresh OP50 to determine live counts. Live counts were processed by a beta version of NemaStudio.ai, a cloud-based data annotation tool, followed by manual annotation (NemaLife, Inc., TX, USA). Infection assays were conducted at 20°C for the indicated time intervals. Statistical analyses were calculated using GraphPad Prism version 9.5.0 (GraphPad Software, San Diego, CA, USA), and *P* values were estimated using the log-rank (Mantel-Cox) test. Statistical tests were performed for each strain/condition compared to the wild-type (PA14) control. For all cases, *P*-values of <0.001 were considered significant.

### *Caenorhabditis elegans* HIF-1 induction microscopy

Images were acquired using a Leica DMi8 fitted with a SpectraX illuminator (Lumencor), an Orca Flash 4.0 v2 sCMOS camera (Hamamatsu), and LasX software (Leica). Relative fluorescence intensity (RFU) was calculated using the LasX relative fluorescence calculator using a 200 × 200-µm square as a background measurement for the fluorescence intensity of the worm. Upregulation of the HIF-1::GFP reporter used to obtain the Fmax (maximum reporter intensity) was captured by inducing hypoxia in the *C. elegans* strain UL1447 (67). The infection for *hif-1* induction was performed according to the shifting assay, apart from using NGM plates, with a 1-day recovery on OP50 prior to imaging. The infection-induced RFU values were normalized to the Fmax. The normalized values were plotted, and *P*-values were generated by Student’s *t*-test using GraphPad Prism version 9.5.0 (GraphPad Software, San Diego, CA, USA).

## SUPPLEMENTAL MATERIAL

Supplemental material msphere.00786-23-s0001.pdf Figures S1-S4 and Tables S1 and S2.

ASM does not own the copyrights to Supplemental Material that may be linked to, or accessed through, an article. The authors have granted ASM a non-exclusive, world-wide license to publish the Supplemental Material files. Please contact the corresponding author directly for reuse.
