# Supplementary material for: Glycerol metabolism impacts biofilm phenotypes and virulence in Pseudomonas aeruginosa via the Entner-Doudoroff pathway
Source: mSphere. 2024 Mar 19;9(4):e00786-23. doi: 10.1128/msphere.00786-23 (PMC11036800; doi:10.1128/msphere.00786-23)
Supplement: Supplemental material — Figures S1-S4 and Tables S1 and S2. [file msphere.00786-23-s0001.pdf]

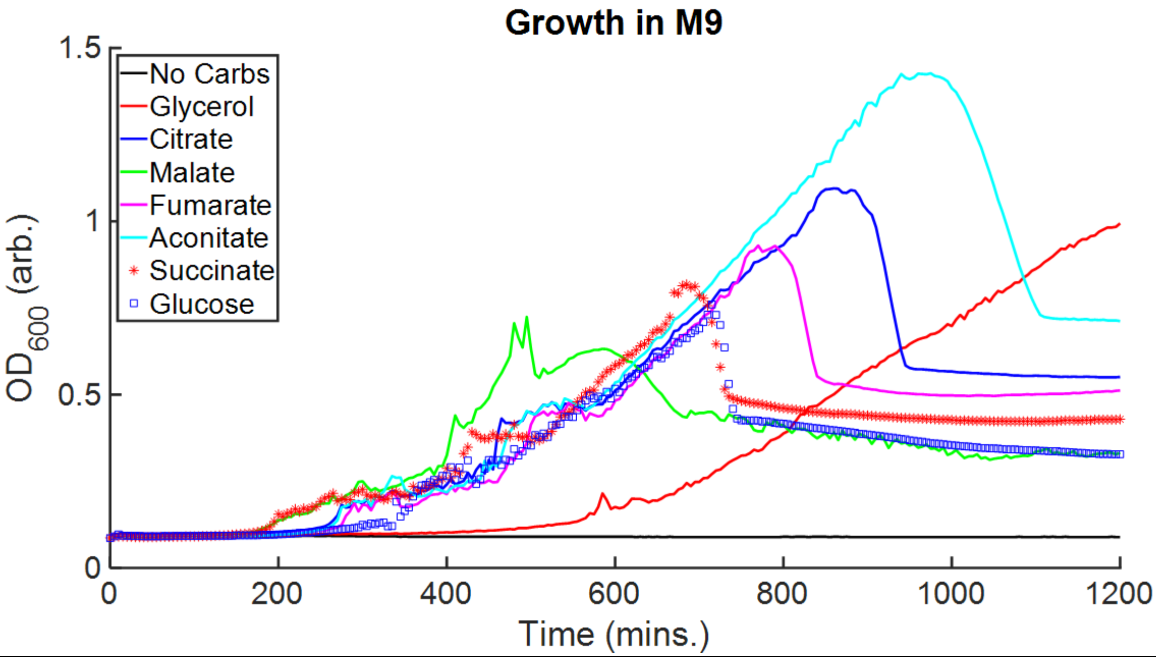

Supporting Figure S1: Growth (OD_{600}) vs. time for *Pseudomonas aeruginosa* PA14 in M9 minimal medium supplemented with no carbon source added (black line), 0.5% v/v glycerol (red line), 7.5 mM citrate (blue line), 7.5 mM malate (green line), 7.5 mM fumarate (magenta line), 7.5 mM aconitate (cyan line), 7.5 mM succinate (red stars), or 3 mM glucose (blue squares). No growth is observed when a carbon source is not present.

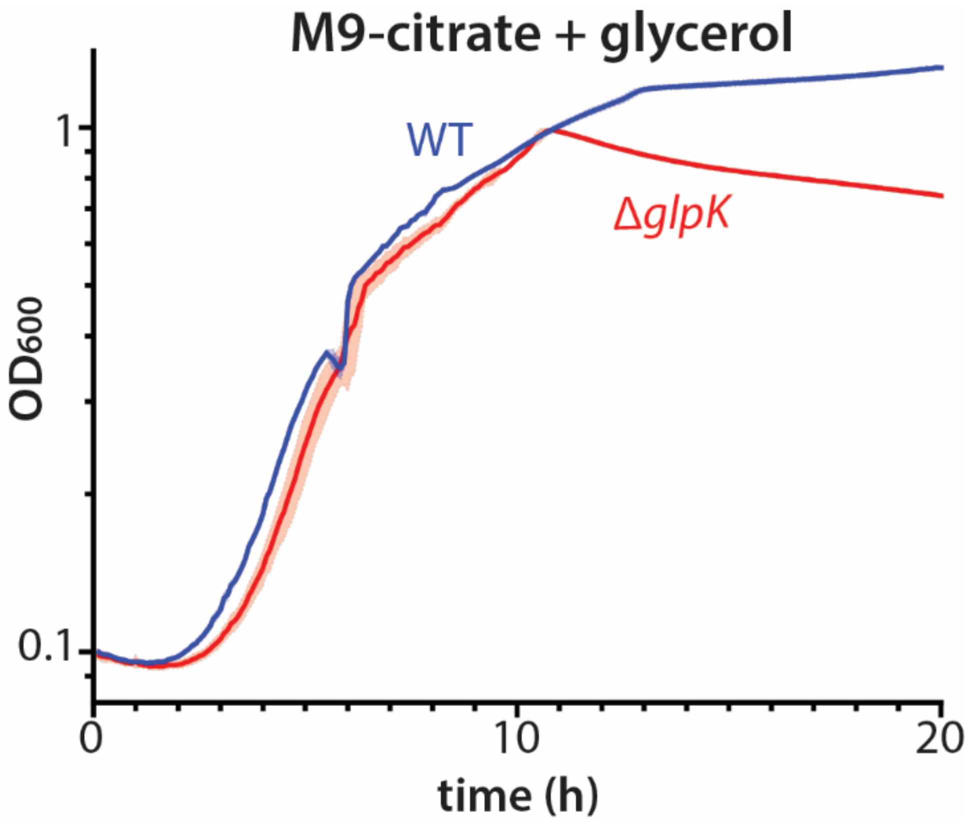

Figure S2

Supporting Figure S2: Growth curves of PA14 (WT) and Δ*glpK* as indicated in M9 supplemented with 17 mM citrate and 34 mM glycerol. The curves shown are averages of 3 biological replicates, each of which is an average of 3 technical replicates. Shading shows standard deviation among biological replicates.

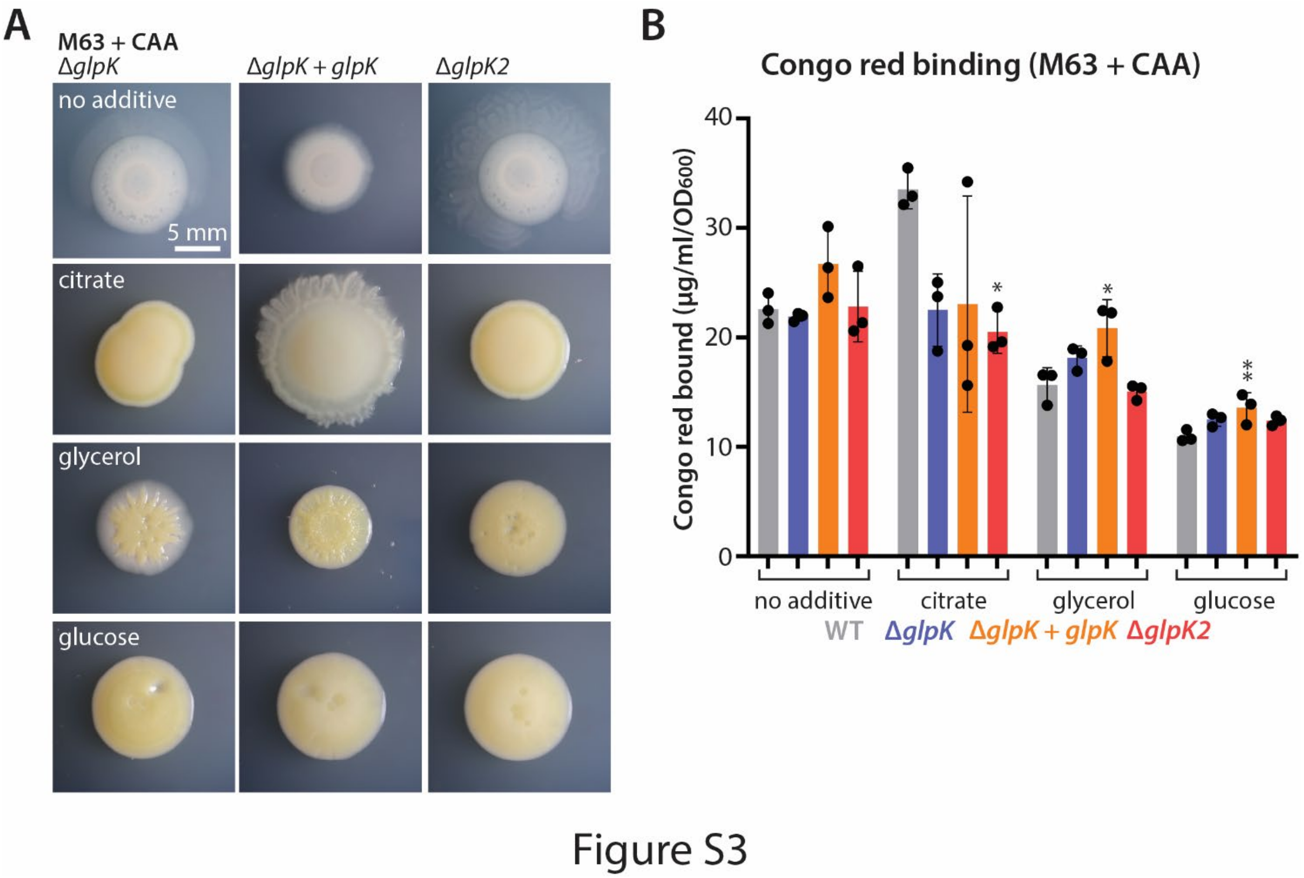

Supporting Figure S3: A. Photographs of representative colonies of the indicated strains grown for 6 days at 25°C on M63-1% agar + 0.2% casamino acids (CAA) agar, either without further supplementation ("no additive") or with 68 mM glycerol, 34 mM citrate, or 34 mM glucose as indicated. **B.** Congo red binding of the indicated strains on the same medium as noted in panel A. The "+ *glpK*" is shorthand for complementation of *glpK* at the *attB* locus. Statistical comparisons used one-way ANOVA followed by Dunnett's multiple comparisons test, using the wild type as the control for each condition. *, $p \leq 0.05$; **, $p < 0.01$.

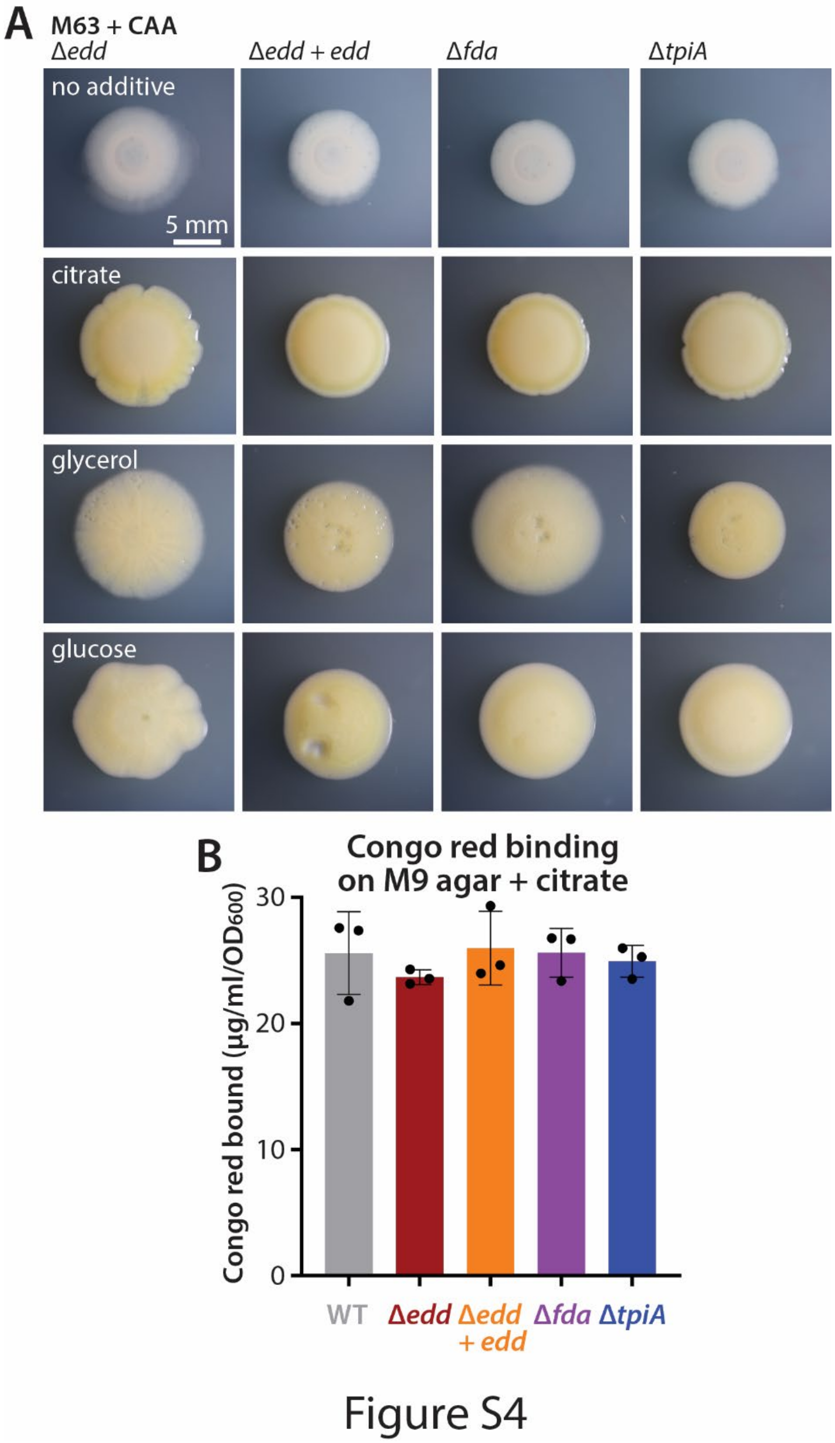

Supporting Figure S4: A. Photographs of representative colonies of the indicated strains grown for 6 days at 25°C on M63-1% agar + 0.2% casamino acids (CAA) agar, either without further supplementation ("no additive") or with 68 mM glycerol, 34 mM citrate, or 34 mM glucose as indicated. **B.** Congo red binding of the indicated strains on M9-1% agar with 34 mM citrate as the only carbon source. The "+ *edd*" is shorthand for complementation of *edd* at the *attB* locus. Statistical comparisons used one-way ANOVA followed by Dunnett's multiple comparisons test, using the wild type as the control for each condition.

Primer name	Sequence
1516 (glpK dwn fw new)	**GGCCACAGCC** CCGGCTGGAAAAAAGCGGTGG
1517 (glpK up R new)	**TTCCAGCCGG** GGCTGTGGCCTTGTGG
1299 (pEX glpK up FW)	**TGCGCACCCGTGGAAATTAATTAAGGTACC GAATTC CATCGCGCTATGGCTG**
1302 (pEX glpK dwn rev)	**TTATACGAGCCGGAAGCATAAATGTAAAGC AAGCTT GATGCCGCCGTCGCTG**
1365 (pEX glpK2 up FW)	**TGCGCACCCGTGGAAATTAATTAAGGTACC GAATTC** TATCGCTACCGAGGC
1366 (glpK2 upR)	**AACGCCGCTCA** GGAAAACCTGACAGATTG
1367 (glpK2 dwn F)	**TCAGGTTTTCC** TGAGCGGCGTTTCGC
1368 (pEX glpK2 dwn rev)	**TTATACGAGCCGGAAGCATAAATGTAAAGC AAGCTT** AGCAGTACCAGCAGCTTG
1574 (pEX fba up FW)	**TGCGCACCCGTGGAAATTAATTAAGGTACC GAATTC** GATCCGACCGCCGGACAAAAG
1575 (fba up REV)	**GAAGGCGCTTAG** GGTTCTCGTATCTCCCAATCGCG
1576 (fba dwn FW)	**GATACGAGAACC** CTAAGCGCCTTCGTTCGAACG
1577 (pEX fba down REV)	**TTATACGAGCCGGAAGCATAAATGTAAAGC AAGCTT** CTGTTCGACCTGCAGCCCTG
1578 (pEX tpi up fw)	**TGCGCACCCGTGGAAATTAATTAAGGTACC GAATTC** GTGATGATGGTCGACCATAC
1579 (tpi up rev)	**GAACAACTC** GAATGCGTACCGTGCATTTTC
1580 (tpi dwn FW)	**GTACGCATTC** GAGTTGTTCGGCATGCCGG
1581 (pEX tpi dwn REV)	**TACGAGCCGGAAGCATAAATGTAAAGC AAGCTT** CAAAGCTGGTGCAAGATTATACGACCG
1566 (pEX edd up FW)	**TGCGCACCCGTGGAAATTAATTAAGGTACC GAATTC** GATCATCGACTGGGTCGCCG
1567 (edd up REV)	**CTATCAGAATC** CATGGCAGATTCTCCTACAGACTATC
1568 (edd dwn FW)	**GAATCTGCCATG** GATTCTGATAGGTCGATGTCCG
1569 (pEX edd dwn rev)	**TTATACGAGCCGGAAGCATAAATGTAAAGC AAGCTT** CTGATTTCATAGAGGGCGAGC

Supporting Table S1: PCR primers used in this study.

***E. coli* strains**			
Strain number	Strain name	Genotype	Source or citation
MTC27	SM10	F- *endA1 hsdR17 supE44 thi-1 λ- recA1 gyrA96 relA1 E. coli* strain for conjugation with *P. aeruginosa*	[1]
MTC569	SM10 pEXG2 Δ*amrZ*	*E. coli* SM10 bearing conjugative plasmid pEXG2 with cassette to delete *amrZ* gene	[2]
SUECO49	SM10 pEXG2 Δ*glpK*	*E. coli* SM10 bearing conjugative plasmid pEXG2 with cassette to delete *glpK* gene	This study
SUECO58	SM10 pEXG2 Δ*glpK2*	*E. coli* SM10 bearing conjugative plasmid pEXG2 with cassette to delete *glpK2* gene	This study
SUECO95	SM10 pEXG2 Δ*fba*	*E. coli* SM10 bearing conjugative plasmid pEXG2 with cassette to delete *fba* gene	This study
SUECO94	SM10 pEXG2 Δ*tpi*	*E. coli* SM10 bearing conjugative plasmid pEXG2 with cassette to delete *tpi* gene	This study
SUECO97	SM10 pEXG2 Δ*edd*	*E. coli* SM10 bearing conjugative plasmid pEXG2 with cassette to delete *edd* gene	This study
OP50	OP50	*E. coli* OP50 strain, used to feed *C. elegans*	Caenorhabditis Genetics Center (CGC)
**Plasmids**			
	pEXG2	Integrative suicide plasmid for *P.*	[3]

		aeruginosa, gentamycin^{R}, with *sacB* sucrose counterselection marker	
	pEXG2 Δ*amrZ*	pEXG2 carrying flanking regions of *amrZ* gene for markerless deletion	[2]
	pEXG2 Δ*glpK*	pEXG2 carrying flanking regions of *glpK* gene for markerless deletion	This study
	pEXG2 Δ*glpK2*	pEXG2 carrying flanking regions of *glpK2* gene for markerless deletion	This study
	pEXG2 Δ*fba*	pEXG2 carrying flanking regions of *fba* gene for markerless deletion	This study
	pEXG2 Δ*tpi*	pEXG2 carrying flanking regions of *tpi* gene for markerless deletion	This study
	pEXG2 Δ*edd*	pEXG2 carrying flanking regions of *edd* gene for markerless deletion	This study

Supporting Table S2: *E. coli* strains and plasmids used in this study.

References

1. Simon R, Priefer U, Pühler A. A Broad Host Range Mobilization System for In Vivo Genetic Engineering: Transposon Mutagenesis in Gram Negative Bacteria. Nat Biotechnol. 1983;1: 784–791. doi:10.1038/nbt1183-784

2. Cabeen MT, Leiman SA, Losick R. Colony-morphology screening uncovers a role for the Pseudomonas aeruginosa nitrogen-related phosphotransferase system in biofilm formation. Molecular Microbiology. 2016;99: 557–570. doi:https://doi.org/10.1111/mmi.13250

3. Rietsch A, Vallet-Gely I, Dove SL, Mekalanos JJ. ExsE, a secreted regulator of type III secretion genes in Pseudomonas aeruginosa. Proc Natl Acad Sci U S A. 2005;102: 8006–8011. doi:10.1073/pnas.0503005102
